# Radiographic quantification of the normal and near-normal coxofemoral conformation in Labrador Retrievers and German Shepherds: a comparative study

**DOI:** 10.1186/s13620-023-00234-z

**Published:** 2023-02-27

**Authors:** Menna A. Nahla, Clifford R. Berry, Ayman A. Mostafa

**Affiliations:** 1 Department of Small Animal Surgery and Radiology, Faculty of Veterinary Medicine, Cairo University, Giza, 12211 Egypt; 2 Diagnostic Imaging, Department of MBS, College of Veterinary Medicine, North Carolina State University, Raleigh, NC 27606 USA

**Keywords:** Radiographic quantification, Normal and near-normal, Coxofemoral conformation, Dogs, Comparative

## Abstract

**Background:**

Canine hip dysplasia (CHD) is a multifactorial disease affecting large breed dogs with associated joint laxity and incongruity that predisposes them to osteoarthritis. The purpose of the study is to objectively compare the conformation of normal and near-normal coxofemoral joints (CFJ_S_) in Labrador Retrievers versus German Shepherds on the extended ventrodorsal radiograph. Investigated groups were categorized as normal and near-normal CFJ_S_ according to the morphometric criteria established by the FCI scoring system. Center-edge (CE) angle, Norberg angle (NA), indices of dorsal AFH coverage width and area, acetabular slope (AS) angle, and inclination angle were determined for each group. CE angle and AS angle were modified from previously described human techniques. The width and area of dorsal AFH coverage were standardized by the corresponding femoral head diameter and area. Variables were compared between groups using an unpaired, two-tailed *t*-test. A Spearman correlation coefficient determined the relationship between selected variables.

**Results:**

In Labradors, CE angle (lateral coverage) and dorsal AFH coverage area index (dorsal coverage) were greater in normal versus near-normal CFJ_S_. In German Shepherds, lateral AFH coverage (CE angle and NA) was greater in normal versus near-normal hip joints; whereas, dorsal AFH coverage did not differ between the two groups. Lateral AFH coverage was greater in normal versus near-normal CFJ_S_ of both breeds. In Labradors, the inclination angle was greater in near-normal versus normal CFJ_S_. Normal CFJ_S_ of Labradors revealed greater lateral and dorsal AFH coverages compared to German Shepherds. Near-normal joints of Labradors showed greater lateral AFH coverage compared to those of German Shepherds; whereas, dorsal AFH coverage did not differ between the two breeds. A steeper acetabular slope angle was noted in normal and near-normal CFJ_S_ of German Shepherds compared to Labrador Retrievers. The inclination angle of near-normal joints was greater in Labrador Retrievers compared to German Shepherds.

**Conclusions:**

Overall, normal and near-normal CFJ_S_ of German Shepherds had lesser AFH coverage and steeper acetabular slope angle compared to Labrador Retrievers. Labrador Retrievers and German Shepherds with CE-angles < 27° and < 21.8°, dorsal AFH coverage width indices < 51 and < 49%, and/or dorsal AFH coverage area indices < 53 and < 50%, respectively, may be consistent with CHD. Thus, the authors would recommend excluding subjects with lower values from breeding. Validating the reported measurements is still warranted.

## Background

Canine hip dysplasia (CHD) is a common orthopaedic problem, accounting for 30% of all orthopaedic diseases [1]. It is a developmental, heritable, multifactorial disorder of the coxofemoral joint (CFJ) with associated joint instability that can eventually lead to painful degenerative joint disease [2, 3]. It primarily affects rapidly growing large breed dogs [4], with German Shepherds, Labrador Retrievers, and Boxers having a high prevalence [5, 6]. Many radiographic techniques and measurements have been developed and widely utilized for evaluating canine hip joints [7–9]. The extended-leg ventrodorsal (VD) pelvic view remains the most used technique for evaluating canine CFJ [10]. Norberg angle (NA) and centre-edge (CE) angle are common radiographic measures used to assess the degree of lateral acetabular femoral head (AFH) coverage in dogs and humans, respectively [11–15]. However, the technique of measuring NA relies on the consideration of both hip joints meaning that it does not accurately represent the hip joint conformation and thus has weaknesses as a solar selection criterion [16, 17]. A recent study that applied the CE angle on the canine CFJ in a modified way from the human technique reported promising results supporting the feasibility of using the modified CE angle to quantify the degree of lateral AFH coverage in dogs and overcome the possible imperfection of NA [18]. The impetus of the current study was the observation that no prior studies have evaluated and compared the degrees of both lateral and dorsal AFH coverage, the steepness of acetabular slope angle, and the inclination angle in different breeds of dogs. Therefore, our main objective was to compare the radiographic quantification of normal and near-normal coxofemoral conformation of Labrador Retrievers and German Shepherds (the most commonly affected breeds with CHD) via assessing the degree of lateral and dorsal AFH coverage and evaluating the steepness of the acetabular slope and the inclination angle. We hypothesised that the AFH coverage, the acetabular slope angle, and the angle of inclination would differ between Labrador Retrievers and German Shepherds and possibly between the 2 groups of coxofemoral joints (normal versus near-normal) within each breed. The long-term aim of the study is to develop a selective breeding strategy that uses parents with healthy coxofemoral joints (phenotypically) in an attempt to decrease the prevalence of CHD among the Labrador Retrievers’ and German Shepherds’ offspring.

## Methods

### Subjects

The retrospective study protocol was approved by the Scientific Committee of the Department of Surgery and Radiology at the Faculty of Veterinary Medicine, Cairo University prior to investigation. Medical records and extended VD pelvic radiographs of adult Labrador Retrievers and German Shepherds with normal and near-normal CFJs were retrieved from the database of the Small Animal Hospital, University of Florida, College of Veterinary Medicine. All enrolled pelvic radiographs were belonging to individuals with no clinical or radiographic signs of orthopaedic diseases. All digitized radiographs were approved in terms of quality and positioning (with parallel femurs and no pelvic tilting) [19, 20] and were sorted out into normal (grade A) and near normal (grade B) categories by board-certified and qualified radiologists (CB and AM). This categorization was carried out according to the morphometric criteria previously established by the Federation Cynologique Internationale (FCI) scoring protocol of CHD [10, 21, 22]. Accordingly, the hip joint was considered normal (grade A) in both Labrador Retrievers and German Shepherds if the CFJ space appeared narrow with sharply margined perfectly parallel articular margins, circumferentially (perfectly congruent joint), and a NA ≥ 105^o^. The near-normal CFJ (grade B) showed sharply margined and nonparallel coxofemoral articular surfaces with associated slightly widened joint space (minimal joint incongruence). The enrolled CFJs were considered near-normal if the NAs were ≥ 105^o^ in Labrador Retrievers and ≤ 105° in German Shepherds. Coxofemoral joints with radiographic evidence of hip dysplasia (grades C to E) were excluded from the current report.

### Radiographic procedures

**Fig. 1:**
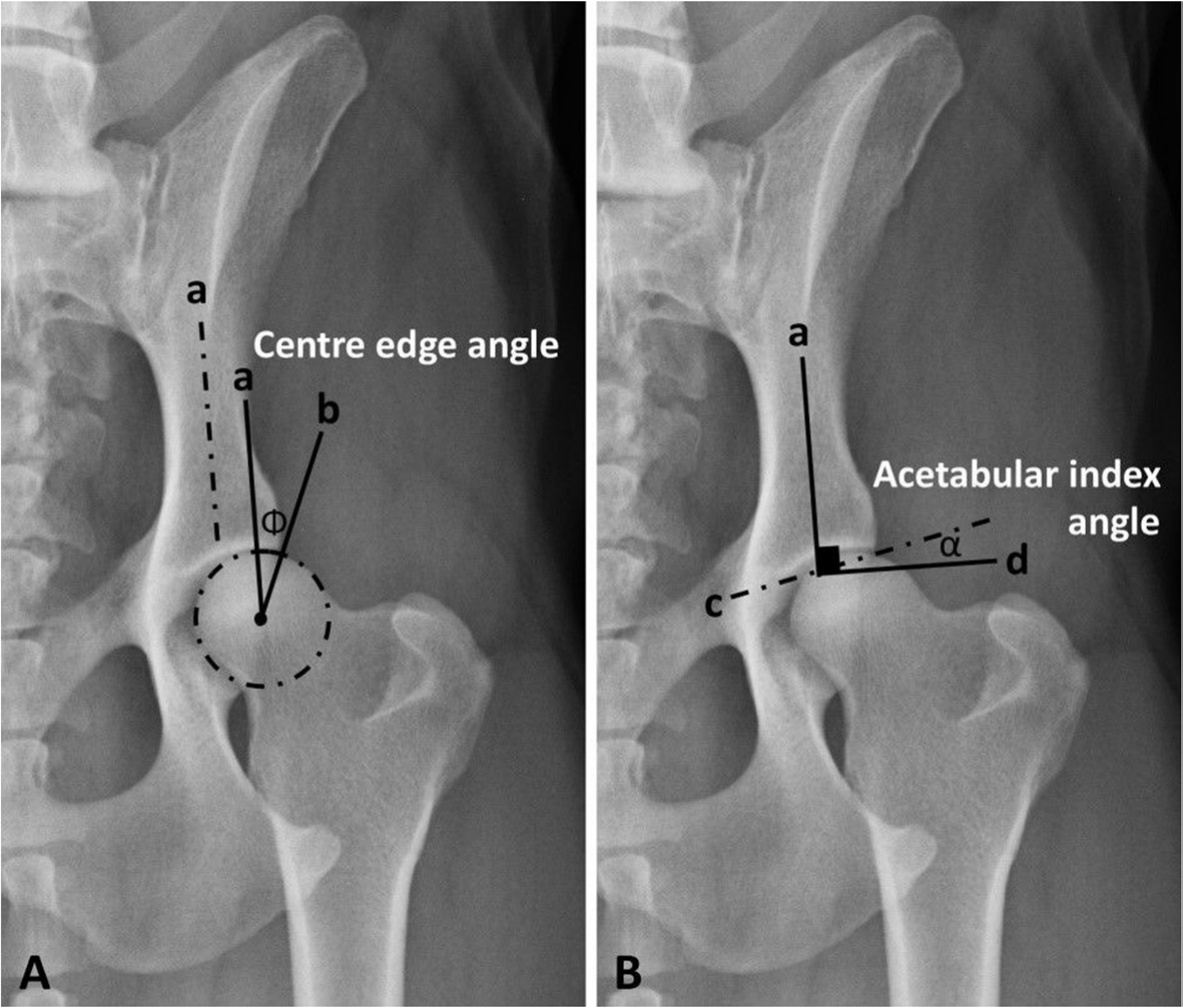
An extended ventrodorsal radiograph of a German Shepherd’s pelvis with a near-normal hip joint showing measurements of (**A**) centre edge angle (Φ) and (**B**) acetabular slope angle (α). *a*, iliac axis; *b*, a line tangential to the lateral acetabular rim, originating from the center of the corresponding femoral head; *c*, a line tangential to the acetabular sourcil; *d*, a line verticle to the long axis of the iliac shaft (*a*)

**Fig. 2:**
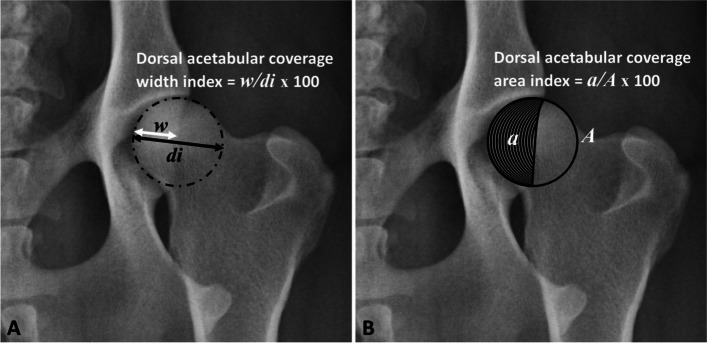
An extended ventrodorsal radiograph of a German Shepherd’s pelvis with a near-normal hip joint showing measurements of (**A**) the width index of the dorsal acetabular femoral head (AFH) coverage and (**B**) the area index of the dorsal AFH coverage. *w*, dorsal AFH coverage width; *di*, femoral head diameter; *a*, dorsal AFH coverage area; *A*, femoral head area

All measurements were carried out blindly on digitized radiographs by the same veterinarian (MN). The reported measurements were performed using freely available medical and radiologic image processing software (ImageJ 1.41/Java 1.6.0_21) with a magnification of 200 as previously established [14, 15]. A best-fit circle outlining the femoral head was initially drawn to precisely locate its center and measure its area. The CE angle, the acetabular slope (AS) angle, and the indices of dorsal AFH coverage width and area were measured as previously published [18] (Figs. 1 and 2) (Table 1). The NA and IA (method B) were measured as previously described by several veterinary studies [14, 15, 23–25] (Table 1).

**Table 1:** Definitions and functions of the modified centre-edge, Norberg, acetabular slope, and inclination angles, and the width and area indices of the dorsal acetabular femoral head (AFH) coverage

**Variable**	**Definition**	**Function**
**Modified centre-edge (CE) angle**	The angle between two lines originating from the femoral head center, a line tangential to the lateral acetabular rim and a second line parallel to the long axis of the body of the corresponding ilium (iliac axis).	Evaluates the degree of lateral acetabular femoral head (AFH) coverage.
**Norberg angle (NA)**	The angle between a line connecting the centers of the femoral heads and a line connecting the center of the femoral head to the corresponding lateral edge of the cranial acetabular rim.	Evaluates the degree of lateral acetabular femoral head (AFH) coverage.
**Acetabular slope (AS) angle**	The angle between a line connecting the lateral and medial extents of the sclerotic cranial acetabular edge and a horizontal line perpendicular to the corresponding iliac axis.	Quantifies the steepness of the cranial acetabular edge (acetabular “sourcil” slope).
**Inclination angle (IA) (Method B)**	The angle formed between a line bisecting the shaft of the femur and the second line bisecting the femoral head/ neck.	Evaluates the proximodistal alignment of the femoral head and neck relative to the corresponding femoral axis.
**Dorsal AFH coverage width index**	The width of the dorsal AFH coverage divided by the diameter of the corresponding femoral head.	Determine the extent of dorsal AFH coverage.
**Dorsal AFH coverage area index**	The area of the dorsal AFH coverage divided by the overall area of the corresponding femoral head.	

### Statistical analysis

Data analysis was performed using commercially available statistical software (Graph-Pad Prism version 8.00, La Jolla, California, United States). Data were proven to be normality distributed using the Kolmogorov-Smirnov test prior to analysis. Means (±SDs) and 95% CIs of all measurements were calculated, and variables of interest were compared between normal and near-normal CFJs for Labrador Retrievers and German Shepherds using an unpaired, 2-tailed *t-*test. A Spearman rank correlation coefficient (*r*_*s*_) was calculated to determine the relationship between selected variables. A *P*-value < 0.05 was considered significant.

## Results

### Subjects

Thirty-seven purebred Labrador Retrievers (74 coxofemoral joints) with radiographically normal (grade A, 23 dogs, 46 joints) and near-normal (grade B, 14 dogs, 28 joints) coxofemoral joints were investigated. As for German Shepherds, 38 purebred dogs (76 coxofemoral joints) with radiographically normal (grade A, 21 dogs, 42 joints) and near-normal (grade B, 17 dogs, 34 joints) coxofemoral joints were investigated.

### Radiographic procedures

#### Labrador Retrievers

The NA did not differ significantly between normal and near-normal coxofemoral joints; however, the CE angle differed significantly (*P* = 0.013) between the two tested groups. As for the measurements utilized to quantify dorsal AFH coverage, the area index was significantly greater (*P* = 0.0003) in normal versus near-normal coxofemoral joints; whereas, the width index did not differ between the two groups. There was a significant increase (*P* = 0.0003) in the mean inclination angle of near-normal coxofemoral joints compared to that of normal joints. No significant difference was identified in the acetabular slope angle between groups.

#### German Shepherds

The measurements utilized to evaluate lateral AFH coverage (NA and CE angle) were greater (*P* < 0.0001) in normal versus near-normal coxofemoral joints. However, the measurements that quantified dorsal AFH coverage (width and area indices) did not differ significantly between the tested groups. Neither the acetabular slope angle nor the inclination angle differed between the two groups. The mean (±SD) values and 95% CIs for all reported radiographic measurements of both Labrador Retrievers and German Shepherds are summarized in Table 2.

**Table 2:** Means (SD) and 95% CIs for the age, body weight, and the radiographic parameters calculating lateral (Norberg and Centre edge angles) and dorsal (width and area indices) acetabular femoral head coverage, acetabular slope angle, and inclination angle for Labrador Retrievers and German Shepherds with normal and near-normal coxofemoral joints (CFJs)

Variable	Labrador Retriever				German Shepherd			
	Normal CFJ (***n*** = 46)		Near-normal CFJ (***n*** = 28)		Normal CFJ (***n*** = 42)		Near-normal CFJ (***n*** = 34)	
	95% CI	Mean (SD)	95% CI	Mean (SD)	95% CI	Mean (SD)	95% CI	Mean (SD)
Age (year)	8.6–10.8	9.7 (2.8)	7.6–9.6	8.6 (2.1)	3.8–6.1	5.0 (3.3)†	2.6–5.3	4.0 (3.6)•
Body weight (kg)	30.0–34.6	32.3 (5.8)	30.6–35.2	32.9 (5.4)	33.4–38.2	35.8 (6.6)†	31.8–37.2	34.5 (6.5)
Norberg angle (degree)	109.3–111.0	110.2 (2.8)	108.3–110.3	109.3 (2.6)	106.8–108.3	107.5 (2.3)†	102.8–103.6	103.2 (1.1)*•
Centre-edge angle (degree)	28.4–29.2	28.8 (1.2)	27.0–28.6	27.8 (2.1)*	26.1–27.1	26.6 (1.6)†	21.8–22.7	22.3 (1.3)*•
Dorsal acetabular coverage width index	0.54–0.57	0.56 (0.05)	0.51–0.56	0.54 (0.06)	0.51–0.55	0.53 (0.05)†	0.49–0.54	0.51 (0.07)
Dorsal acetabular coverage area index	0.58–0.61	0.60 (0.05)	0.53–0.57	0.55 (0.06)*	0.53–0.56	0.55 (0.05)†	0.50–0.55	0.53 (0.07)
Acetabular slope angle (degree)	6.7–9.1	7.9 (4.1)	6.9–9.3	8.1 (3.0)	11.5–14.6	13.1 (4.9)†	11.2–14.3	12.7 (4.5)•
Inclination angle (degree)	128.6–132.7	130.6 (6.8)	134.2–138.7	136.5 (5.8)*	128.7–132.1	130.4 (5.4)	128.2–132.1	130.2 (5.6)•

^*^Within a variable, the mean is significantly (*P* < 0.05) different from the mean for the normal CFJs within the same breed group

^†^Within a variable, the mean is significantly (*P* < 0.05) different from the mean for the normal CFJs of the other breed group

^•^Within a variable, the mean is significantly (*P* < 0.05) different from the mean for the near-normal CFJs of the other breed group

#### Labrador Retrievers versus German Shepherds

**Fig. 3:**
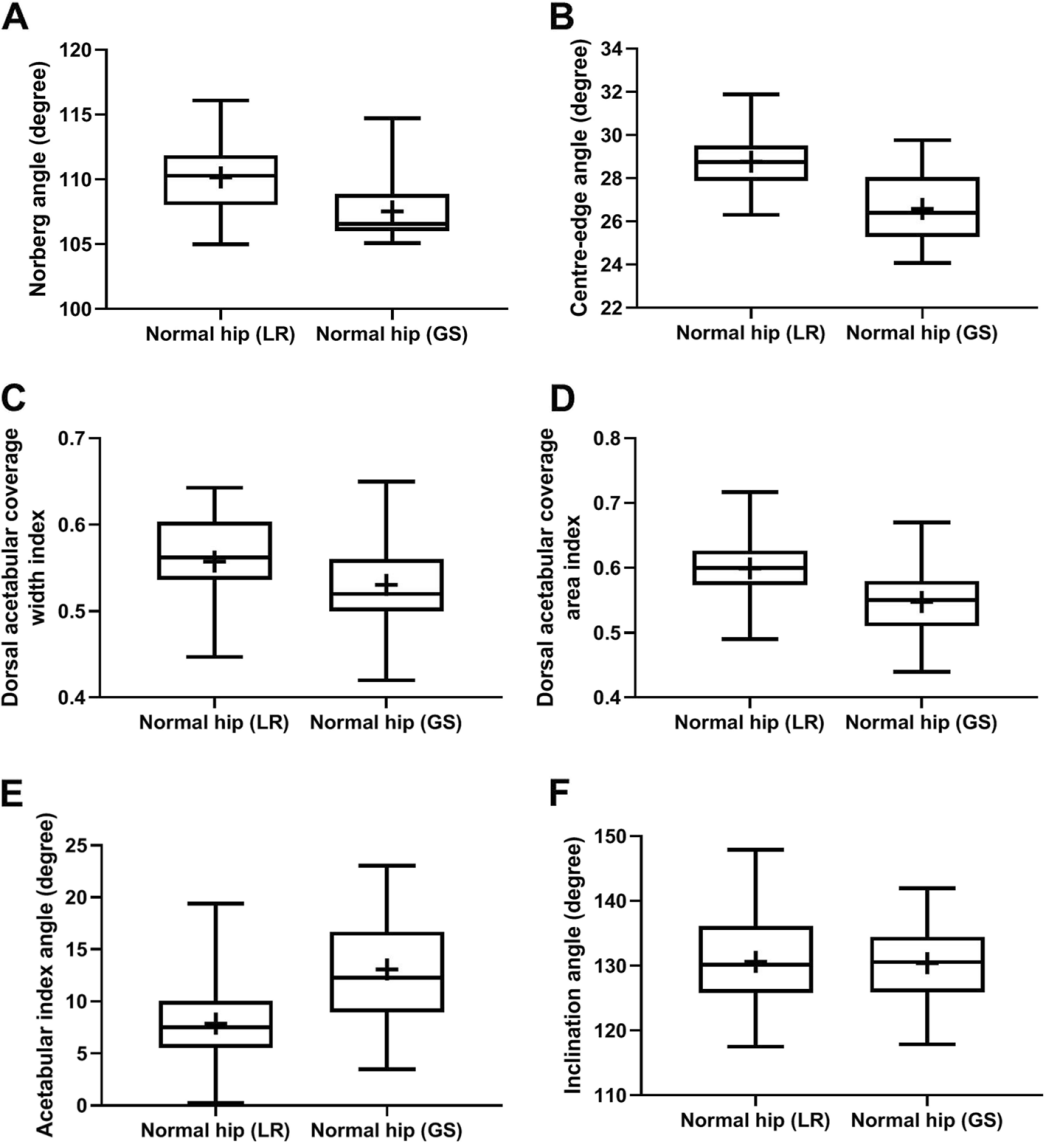
Box-and-whisker plots of Norberg angle (**A**), center-edge angle (**B**), dorsal acetabular coverage width index (**C**), dorsal acetabular coverage area index (**D**), acetabular slope angle (**E**), and inclination angle (**F**) for normal coxofemoral joints of Labrador Retriever (LR) and German Shepherd (GS). Boxes and whiskers represent the 25th to 75th percentiles and ranges, respectively; the lines and crosses within boxes represent the medians and means, respectively

**Fig. 4:**
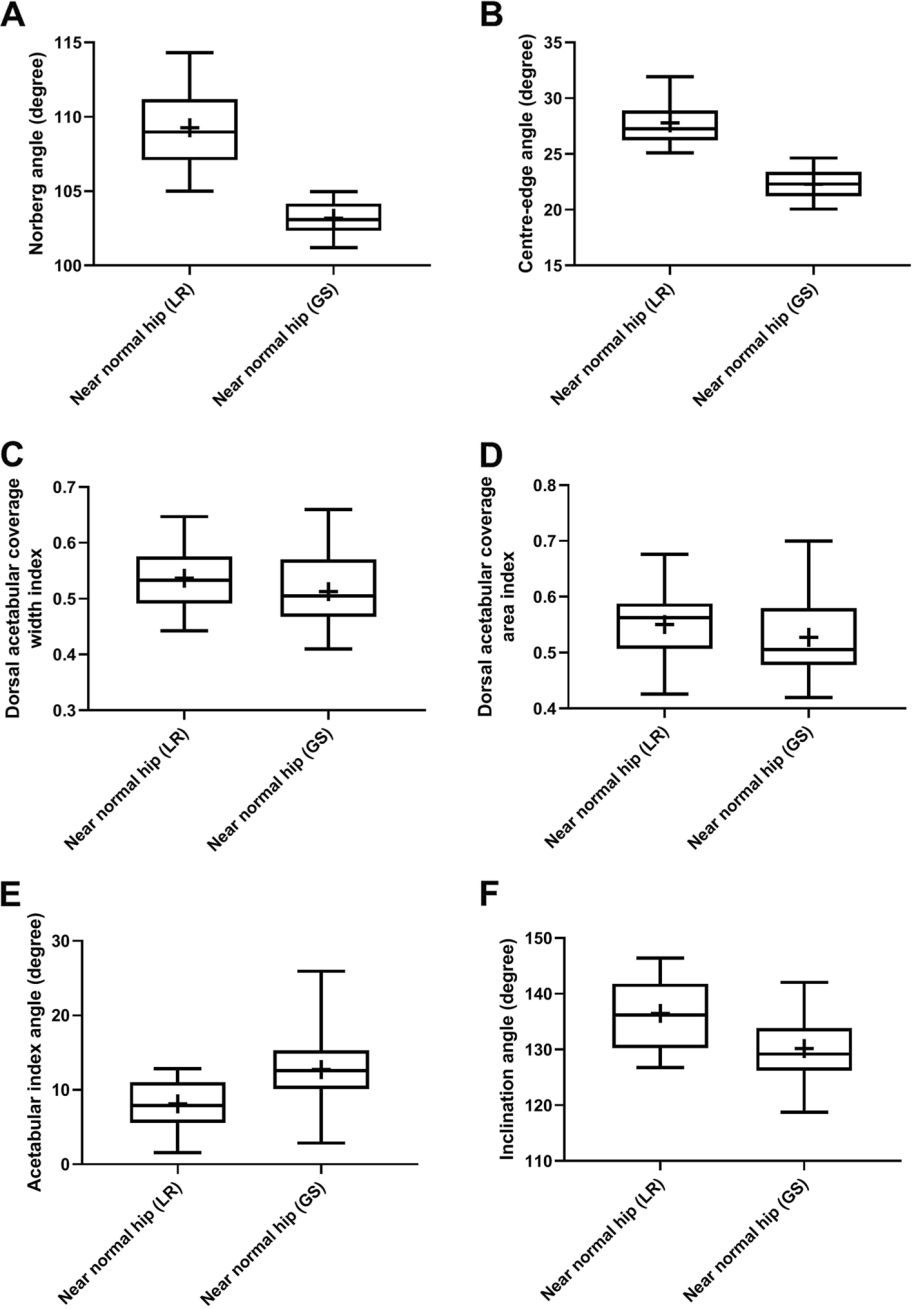
Box-and-whisker plots of Norberg angle (**A**), center-edge angle (**B**), dorsal acetabular coverage width index (**C**), dorsal acetabular coverage area index (**D**), acetabular slope angle (**E**), and inclination angle (**F**) for near-normal coxofemoral joints of Labrador Retriever (LR) and German Shepherd (GS). Boxes and whiskers represent the 25th to 75th percentiles and ranges, respectively; the lines and crosses within boxes represent the medians and means, respectively

Greater (*P* < 0.0001) lateral and dorsal AFH coverages were evidenced in normal coxofemoral joints of Labrador Retriever compared to those evidenced in normal German Shepherd joints (Fig. 3). Near-normal hip joints of Labrador Retrievers showed greater (*P* < 0.0001) lateral AFH coverage compared to those of German Shepherds; whereas, dorsal AFH coverage did not differ between the two breeds with near-normal joints (Fig. 4). Lateral AFH coverage was greater (*P* < 0.0001) in normal versus near-normal coxofemoral joints of both breeds.

## Discussion

The results of the current study can be summarized as follows: (1) CE angle differed between normal and near-normal hip joints of German Shepherds and Labrador Retrievers. CE angles < 27° and < 21.8° in Labrador Retrievers and German Shepherds, respectively, would suggest a lack of optimum lateral AFH coverage and possible joint incongruence; (2) NA differed between normal and near-normal hip joints of German Shepherds; however, it did not differ between the two groups of Labrador Retrievers; (3) dorsal AFH coverage did not differ between normal and near-normal joints of German Shepherds; however, dorsal AFH coverage area index showed better coverage in normal versus near-normal CFJs of Labrador Retrievers; (4) lateral and dorsal AFH coverages were greater in normal joints of Labrador Retrievers compared to those calculated for normal German Shepherds’ CFJs; (5) near-normal coxofemoral joints of Labrador Retrievers showed a relatively greater inclination angle compared to normal joints; and (6) there was no difference in the acetabular slope angle between normal and near-normal joints in both dog breeds; however, steeper angles were identified in German Shepherds’ normal and near-normal CFJs.

Regarding the measures utilized to quantify the degree of lateral AFH coverage, NA did not differ between normal and near-normal hip joints of Labrador Retrievers. This may be attributed to the selected values of NA (≥ 105°) for near-normal joints that were set in our study based on the conventional FCI criteria [10, 21, 22]. This may also explain why the mean NA of our near-normal group (109.3°) differed from those (105.9° and 105.7°) reported by other two veterinary literature [22, 26]. However, the mean NA of our enrolled normal coxofemoral joints (110.2°) was approximately consistent with those (108.4° - 108.8°) reported by previous veterinary literature [22, 27]. In German Shepherds, the means NA of our enrolled coxofemoral joints in group A (107.5°), and group B (103.2°) were relatively consistent with those (group A, 108.5°, 106.9°; group B, 105.85°, 104.6°) reported by previous veterinary literature [22, 28]. However, the means CE-angle of the tested groups (A and B) identified in the present study for Labrador Retrievers (28.8°, 27.8°, respectively) and German Shepherds (26.6°, 22.3°, respectively) differed from those (16.91°, 12.55°, respectively) reported by Meomartino and colleagues in 2002. This relative variation may be related to utilizing the iliac axis in the current study instead of the longitudinal axis used in the previous report to measure the CE angle [22]. Another explanation of such a relative variation could be the different radiographic projection (DAR) utilized by Gaspara and colleagues in 2016 [29]. Nevertheless, the normal values of the CE angle identified in our Labrador Retriever (≥ 27°) and German Shepherd (≥ 21.8) populations were consistent with that (≥ 25°) reported in human literature [30–32], despite the species variability. This may indicate the feasibility of utilizing the corresponding iliac axis instead of using an absolute long axis which may not be realistic in the radiographic examination of an animal pelvis [15, 31]; Even though human and dog anatomy and biomechanics differ.

As for the measures utilized to quantify the degree of dorsal AFH coverage, the area indices reported in our tested groups (A and B) of Labrador Retrievers (60 and 55%, respectively) were relatively consistent with those (59.5 and 54.9%, respectively) reported by Tomlinson and colleagues in 2000 [28]. In German Shepherds, the indices of dorsal acetabular coverage area reported in our tested groups (A and B) (55 and 53%, respectively) were relatively in agreement with those reported by Tomlinson and Johnson (59.4, 54.1%, respectively) [28]. The median indices of dorsal AFH coverage width and area reported in our near-normal groups of Labrador Retrievers (54 and 55%, respectively) and German Shepherds (51 and 53%, respectively) agreed with the median values of linear and surface acetabular overlap (52 and 54%, respectively) previously reported by a study performed on a wide variety of large breed dogs [8]. This agreement could be attributed to the similar measurement procedures performed in the two reports, regardless of the enrolled breeds. Unlike the width index, the area index differentiated between normal and near-normal groups of Labrador Retrievers. This may be because the area index determines the overall dorsal AFH coverage area, not just the corresponding width index. The inability of the indices of dorsal AFH coverage (width and area) to differentiate between normal and near-normal coxofemoral joints of German Shepherds may refer to the relative similarity of dorsal acetabular coverage among these groups.

The acetabular slope angle represents the steepness of the acetabular ‘sourcil’ slope. The inability of the acetabular slope angle to distinguish normal from near-normal CFJ in both Labrador Retrievers and German Shepherds could be related to the excessive steepness of this angle that was previously identified in dysplastic rather than non-dysplastic joints [18]. In the present study, the steeper acetabular slope angle identified in German Shepherds with normal and near-normal CFJs compared to that calculated for Labrador Retrievers (*P* < 0.0001) was consistent with the relatively less AFH coverage associated with German Shepherds’ joints. Our reported acetabular slope angles calculated for normal and near-normal CFJs of Labrador Retrievers (7.9^o^ and 8.1^o^, respectively) and German Shepherds (13.1 and 12.7, respectively) were relatively in agreement with the previous reports (8.6^o^ and 7.8^o^ [18], and 7.1^o^ and 11.6^o^ [22], ,respectively). Thus, acetabular slope angles > 8.1^o^ in Labrador Retrievers or > 12.7^o^ in German Shepherds could be associated with canine hip dysplasia. These findings are in agreement with the recent study by Mostafa and colleagues in 2022, in which, acetabular slope angles >11^o^ were evidenced in canine hip dysplasia [18]. Interestingly, these values are relatively consistent with humans’ angles, as hip joints with acetabular slope angles >13^o^ were consistent with human hip dysplasia (33), despite the substantial inter-species variation between humans and dogs in their standing angles and the natural load applied on the corresponding acetabulum of each species [15, 33–36]. Thus, unlike humans, dogs may experience a greater natural load on the dorsum of the acetabulum than that on the acetabular slope [18]. Therefore, radiographic assessment of both dorsal and lateral AFH coverages, as well as, the acetabular slope angle are strongly recommended by the authors during the routine screening program for CHD. The means inclination angle measured for our normal CFJs of Labrador Retrievers (130.6°) and normal and near-normal CFJs of German Shepherds (130.4° and 130.2°, respectively) were consistent with the normal values (129.4°- 130.6^o^) previously reported in large breed dogs [18, 23, 24]. The inclination angle failed to differentiate between normal and near-normal CFJs of German Shepherds; however, increased inclination (*P* = 0.0003) was evidenced in near-normal compared to normal joints of Labrador Retrievers. Nonetheless, the angle of inclination showed a nonsignificant difference between healthy and dysplastic joints in previous veterinary literature [18, 24, 25].

The reproducibility of our reported radiographic measurements was not evaluated in the current study; therefore, a future investigation evaluating intra- and inter-observer repeatability is recommended by the authors to validate them. Another limitation was the lack of assessment of hip joint laxity via calculating the distraction index (PennHip DI). This may limit the efficacy of the radiographic determination of AFH coverage in our suggested selective screening protocol, as evaluation of joint laxity would exclude additional individuals from the breeding pool [29]. Therefore, a future clinical and radiographic investigation may be warranted on Labrador Retrievers, German Shepherds, and other large breed dogs without and with hip dysplasia. The authors of the current and previous recent studies would strongly recommend including both normal and near-normal coxofemoral joints in the breeding strategy to overcome the possibility of breed extinction over the decades [18]. Our recommendation may differ from that of Flückiger’s report who suggested breeding dogs with mildly dysplastic hips but with certain restrictions [37]. The authors hypothesized that prohibiting dogs with mildly dysplastic hip joints from breeding would reduce the heritability of the disease to the offspring, thereby reducing the prevalence of the disease [18].

## Conclusions

Centre-edge angle differed significantly between normal and near-normal hip joints of both German Shepherds and Labrador Retrievers. Normal and near-normal CFJs of German Shepherds showed lesser AFH coverage and steeper acetabular slope angle compared to the degree of the AFH coverage and the slope of the acetabular slope sourcil of normal and near-normal joints of Labrador Retrievers. No significant difference in the acetabular slope angle was identified between normal and near-normal CFJs of both Labrador Retrievers and German Shepherds. Near-normal CFJs of Labrador Retrievers revealed a relatively greater inclination angle compared to normal joints. Labrador Retrievers and German Shepherds with CE-angles < 27° and < 21.8°, dorsal AFH coverage width indices < 51 and < 49%, and/or dorsal AFH coverage area indices < 53 and < 50%, respectively, may be consistent with dysplastic hip joints. The authors would therefore suggest that consideration be given to eliminating subjects with lower values from breeding. Further investigation is however still warranted to validate the reported radiographic measurements.

## Data Availability

The data sets supporting our results are included in the article. Row data are available upon request to any of the authors (AM: aymostafa@cu.edu.eg; CB: crberry3@ncsu.edu).

## Abbreviations

AFH: Acetabular femoral head
AS: Acetabular slope
CE: Centre edge
CFJ: Coxofemoral joint
CHD: Canine hip dysplasia
FCI: Federation Cynologique Internationale
IA: Inclination angle
NA: Norberg angle
VD: Ventrodorsal
